# Neonatal mortality risk assessment using SNAPPE- II score in a neonatal intensive care unit

**DOI:** 10.1186/s12887-019-1660-y

**Published:** 2019-08-13

**Authors:** Dipak Muktan, Rupa R. Singh, Nisha K. Bhatta, Dheeraj Shah

**Affiliations:** 1 Department of Pediatrics, B.P, Koirala Institute of Health Sciences (BPKIHS), Dharan, Nepal; 2 Department of Pediatrics, University College of Medical sciences, New Delhi, India

**Keywords:** Illness severity score, Neonate, Validation

## Abstract

**Background:**

There are many scoring systems to predict neonatal mortality and morbidity in neonatal intensive care units (NICU). One of the scoring systems is SNAPPE-II (Score for Neonatal Acute Physiology with Perinatal extension-II). This study was carried out to assess the validity of SNAPPE-II score (Score for Neonatal Acute Physiology with Perinatal Extension-II) as a predictor of neonatal mortality and duration of stay in a neonatal intensive care unit (NICU).

**Methods:**

This prospective, observational study was carried out over a period of 12 months from June 2015 to May 2016. Two hundred fifty five neonates, who met the inclusion criteria admitted to NICU in tertiary care hospital, BPKIHS Hospital, Nepal were enrolled in the study and SNAPPE-II score was calculated. Receiver Operating Characteristic (ROC) curve was constructed to derive the best SNAPPE-II cut-off score for mortality.

**Results:**

A total of 305 neonates were admitted to NICU over a period of one year. Among them, 255 neonates fulfilled the inclusion criteria. Out of 255 neonates, 45 neonates (17.6%) died and 210 were discharged. SNAPPE-II score was significantly higher among neonates who died compared to those who survived [median (IQR) 57 (42–64) vs. *22* (14–32), *P* < 0.001]. SNAPPE II score had discrimination to predict mortality with area under ROC Curve (AUC): 0.917 (95% CI, 0.854–0.980). The best cut - off score for predicting mortality was 38 with sensitivity 84.4%, specificity 91%, positive predictive value 66.7% and negative predictive value 96.5%. SNAPPE II score could not predict the duration of NICU stay (*P* = 0.477).

**Conclusion:**

SNAPPE- II is a useful tool to predict neonatal mortality in NICU. The score of 38 may be associated with higher mortality.

## Background

Survival of the newborns admitted to the NICUs does not depend only on birth weight and gestational age, but also on other perinatal factors and physiological parameters, particularly those related with severity of their diseases [1–6].

Scoring systems have been developed and used to assess the severity of the illness and to predict the mortality, morbidity and prognosis of neonates in neonatal intensive care units (NICU). Birth weight, gestational age and APGAR score were the only parameters assessed previously to predict mortality and morbidity. However, the association between mortality prediction and these three factors were not much accurate [6–8].

In 1993, Richardson et al. [5] had formulated the physiology-based score; score for Neonatal Acute Physiology (SNAP), which contains 34 parameters for neonates of all birth-weights and validated it as a predictor of mortality and morbidity [3–5]. They made this score easier by reducing the number of parameters to six. To this score, three more perinatal variables namely birth weight, APGAR scores and small for gestational age (SGA) status were added and renamed it as SNAP II with Perinatal Extension (SNAPPE-II) score [7].

Data validating SNAPPE II score from Nepal are lacking. As the clinical profile of neonates and their outcomes may be different in our scenario, we aimed to assess the validity of this score to predict mortality and duration of NICU stay in a resource poor NICU set-up of Nepal. This may help in prioritizing the treatment of sick newborns as well as counselling of their parents about disease severity.

## Methods

This prospective, observational study was carried out during the period from June 2015 to May 2016 at NICU in a tertiary care hospital of eastern Nepal. All newborns admitted to NICU were included in the study. Newborns who died or were discharged in < 24 h after admission, those with congenital malformations incompatible with life, those neonates who did not require ABG (Arterial blood gas analysis) or catheterization, home deliveries with unknown APGAR score and those discharged against medical advice were excluded from our study. Informed consent from parents was taken before conducting this study then participants were enrolled consecutively. This study was approved by the ethical committee of the hospital.

The SNAPPE-II score was calculated on the basis of recommended physiological and clinical factors [7], evaluated prospectively within the first 12 h of admission after stabilization. Noninvasive mean blood pressure in (mmHg) was measured with the use of appropriate cuff size in left or right arm via vital sign monitor (Nihon Kohden Corporation, japan). Temperature was measured in axilla using commercially available mercury thermometer (35 to 42 °C) keeping thermometer for 3 min in axilla. Serum pH and PaO2/FiO2 was calculated by arterial blood gas analysis (ABG) using blood gas and electrolytes analyzer ABL 800 basic (Radiometer, Denmark) available in our NICU. All types of neonatal seizure were included in this score. Birth-weight of inborn neonates was measured by electronic weighing machine (Hardik Meditech, Delhi, India) (±5 g error) without clothing. Birth-weight of outborn neonates was recorded from their details mentioned on referral slips. Urine output (ml/kg/hr) was measured using Pediatric urine collecting bag or by catheterization. Modified Ballard score was used to assess the gestational age. Lubchenco’s [9] intrauterine growth chart was used for classification as small for gestational age as birth-weight < 10th percentile for gestational age. Neonates were treated as per hospital protocols and they were discharged from NICU as per standard NICU protocol.

### Statistical analysis

Data were entered in MS excel and coded where necessary. SPSS version 20.0 was used for data analysis. Comparison between survivors and non-survivors was performed using Mann-Whitney test. Chi-square test was used for qualitative variables. The power of SNAPPE II score to predict the neonatal mortality was evaluated by means of Receiver Operating Characteristics (ROC) curve. Optimal cut-off score to predict mortality was determined by visual inspection of the curve at a level that combined maximum sensitivity and optimal specificity. Positive predictive values and negative predictive values were calculated for different cut-off scores. *P* values less than 0.05 was considered as statistically significant.

## Results

A total of 305 neonates were admitted to NICU over a period of one year (June 2015 to May 2016). Among them, 35 neonates were excluded who did not meet the inclusion criteria. Two hundred seventy neonates were enrolled in the study of which 15 neonates left against medical advice (LAMA). Among 255 neonates completing the study, 92 (36.1%) were preterm and 163 (63.9%) were term neonates. Mean (SD) birth-weight was 2422.9 (858.2) g and mean (SD) gestational age was 36.8 (0.2) weeks. Out of 255 neonates, 45 (17.6%) died and 210 were discharged. Neonates with SNAPPE II score 40 to 60, mortality rate was 36.7%, score of ≥40 had mortality rate of 55.1% and score of ≥60 had 100% mortality. General characteristics of neonates admitted to NICU have been shown in Table 1. The median (IQR) SNAPPE II score was significantly higher in the babies who died in comparison to those who survived [57 (42–64) vs. 22(14–32), *P* < 0.001]. Average duration of NICU stay was 4 days. There was no significant correlation between SNAPPE II score and duration of NICU stay (*P* = 0.477).

**Table 1:** General characteristics of the neonates admitted in NICU

Characteristics	*n* = 255
Gender, n (%)	
Male	175 (69%)
Female	80 (31%)
*Mean Gestational age,* mean (SD) week	36.8 (0.2)
Gestational age, n (%)	
Term	163 (63.9%)
Preterm	92 (36.1%)
*Mean Birth weight,* mean (SD) gram	2422.9 (858.2)
Birth weight n (%)	
< 1000 g	8 (3.1%)
1000 g to 2500 g	102 (40%)
> 2500 g	145 (56.9%)
Outcome, n (%)	
Discharged	210 (82.4%)
Expired	45 (17.6%)
SNAPPE II score, mortality (%)	
≥ 40	55.1%
40–60	36.7%
≥ 60	100%
SNAPPE II score ≥ 38	
Sensitivity	84.4%
Specificity	91%
Positive predictive value	66.7%
Negative predictive value	96.5%

**Fig. 1:**
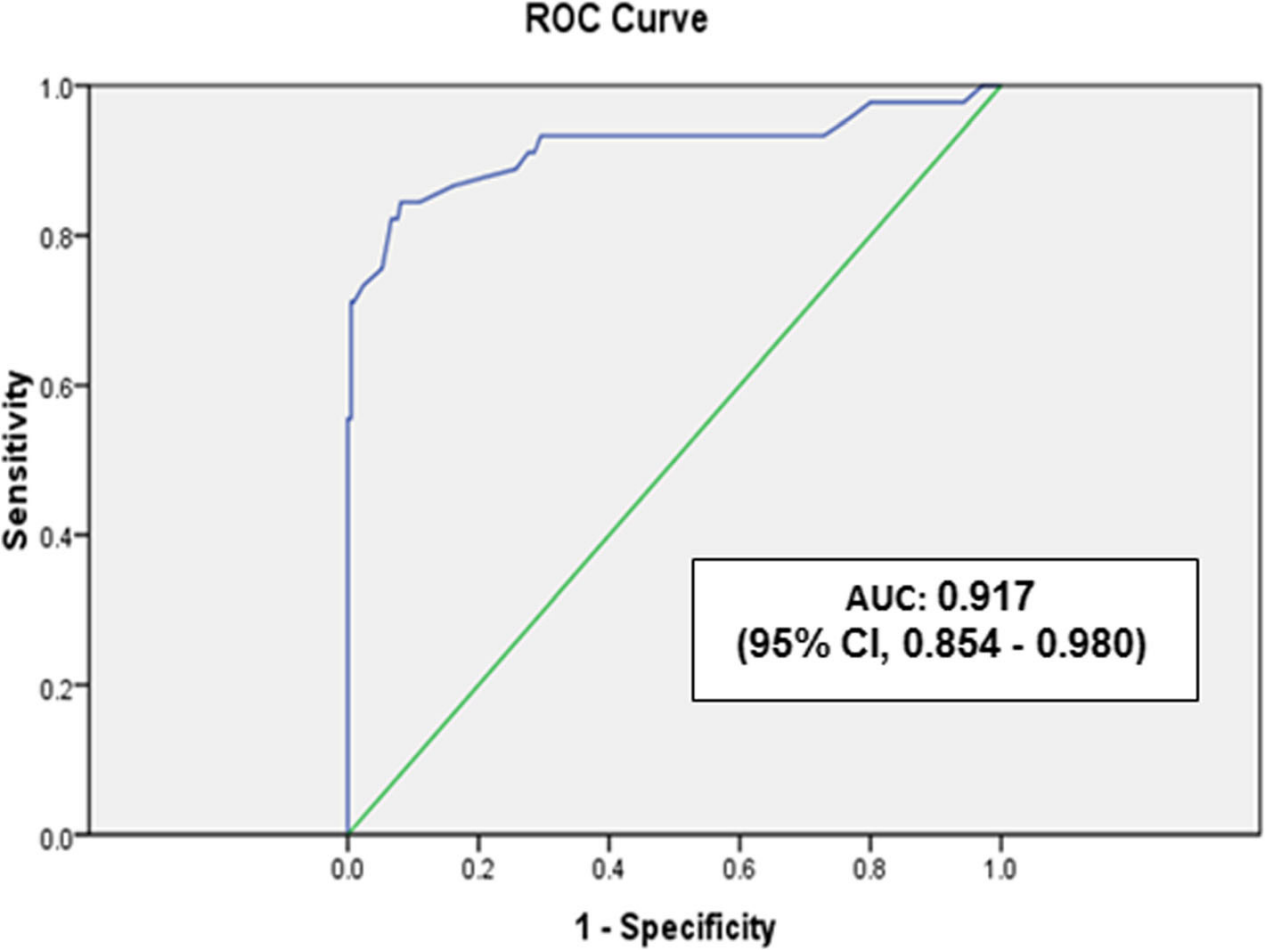
Receiver operating characteristics curve (ROC) for SNAPPE-II score for prediction of mortality

Area under curve (AUC) in ROC curve was 0.917 [95% CI 0.854–0.980] as shown in Fig. 1, which validates the utility of SNAPPE II score to predict neonatal mortality in NICU. The best cut-off SNAPPE II score in predicting overall mortality was 38. Sensitivity, specificity, positive and negative predictive value of score ≥ 38 in estimating overall mortality were 84.4, 91, 66.7 and 96.5% respectively.

## Discussion

The present study documented that the SNAPPE II score of the neonates who died in the NICU was higher than in those who survived. The higher the score of SNAPPE- II, the higher was the mortality risk of neonates. SNAPPE II score of ≥38 was the best to predict mortality with sensitivity 84.4%, specificity 91%, positive predictive value (PPV) 66.7% and negative predictive value (NPV) of 96.5%. There was no significant correlation between SNAPPE II score and duration of NICU stay.

This result supports the study done by original author Richardson et al. (AUC 0.91) [10], Zupanic et al. (AUC 0.90) [11] and Mia et al. [12] in Soetomo Hospital, Indonesia in which AUC was 0.863. In studies conducted in a tertiary care hospital, Indonesia [12] (score of ≥40), in a general pediatric hospital in Paraguay [13] (score of ≥40), Niranjan et al. in India [14] (score of ≥37) & in Indira Gandhi Institute of Child Health, India [15] (score of ≥37) were all associated with higher mortality which is similar to our results. But in contrast to our results, studies conducted in a hospital of indonesia [16] (with a score of ≥51), by Ucar et al. [17], (score of ≥33), Dammann et al. [18], (a score of ≥30) were associated with high mortality. In two studies done in India by Niranjan et al. [14] and (Harsha & Archana) [15] with cut-off score of ≥37 in both studies, Sensitivity (84.4% vs. 76.1% & 76.9%), specificity (91% vs. 87.1% & 87.9%) and NPV (96.5% vs. 52.6%) were higher in our study than these two studies. But positive predictive value in our study was less (66.7 vs. 95. 3%). Variation in the cut-off score and discrimination might be due to the factors affecting the score such as diseases, severity of illness, quality of care in NICU etc. There was no significant correlation between SNAPPE II score and duration of NICU stay (*P* = 0.477). But SNAPPE II score had positive correlation with duration of NICU stay as correlation coefficient was r = 0.045 which is similar to a study done by Harsha & Archana [15] in India where *P* = 0.255 for duration of NICU stay. Other studies also reported similar findings [19, 20].

All newborns who were born at home and those neonates who left NICU against medical advice were excluded from the study. Birth-weight and Apgar score of outborn neonates were taken from referral card. These were the limitations of this study.

Thus, SNAPPE II score is a useful tool to asess the severity of illness and prognosis. These findings can be implicated in NICU routinely to know the most critical newborn for prioritizing the management and for the purpose of counselling the parents. This score might also be used to compare the effectiveness of various NICU across the country which will help to improve the facilities provided by different NICUs.

## Conclusion

SNAPPE II score can be used to predict the severity of diseases and associated mortality and may help in prioritizing the treatment of sick newborns as well as counselling of their parents about disease severity. We conclude that SNAPPE II scoring system may be a useful tool to predict neonatal mortality in resource poor NICU setting.

## Data Availability

Available upon reasonable request to corresponding author.

## Abbreviations

ABG: Arterial blood gas analysis
AUC: Area under ROC Curve
NICU: Neonatal intensive care unit
ROC: Receiver Operating Characteristic
SNAP: Score for Neonatal Acute Physiology
SNAPPE-II: Score for Neonatal Acute Physiology with Perinatal Extension II
